# Non-chemical, chemical, and biochemical, endocrine disruptors: biphasic health effects and pathophysiological insights

**DOI:** 10.3389/fendo.2026.1813234

**Published:** 2026-05-08

**Authors:** Nuriye Nuray Ulusu

**Affiliations:** 1 Department of Medical Biochemistry, Koç University, School of Medicine, Istanbul, Türkiye; 2 Koç University Research Center for Translational Medicine (KUTTAM), Istanbul, Türkiye

**Keywords:** artificial-light-at-night-induced endocrine disruption, chemical or biochemical endocrine disruptors, electromagnetic field-induced endocrine disruption, endocrine metabolism, noise-induced endocrine disruption, thermal stress-induced endocrine disruption

## Abstract

Endocrine-disrupting chemicals (EDCs) interfere with hormone synthesis, signaling, and metabolism, leading to reproductive, metabolic, and neuroendocrine dysfunction. Non-chemical environmental endocrine stressors, including electromagnetic fields, noise, artificial light at night, and thermal stress, can disrupt endocrine homeostasis, alter neuroendocrine and hormonal function, affect signaling pathways, oxidative stress responses, and circadian rhythms. Unlike classical EDCs, which primarily exert their effects by direct binding to hormone receptors and metabolic enzymes, non-chemical endocrine disruptors predominantly act through central regulation of hypothalamic-pituitary-organ axes. EDCs, especially their biochemically active secondary metabolites, disrupt endocrine homeostasis by directly binding target hormone receptors or enzymes and then interacting with the hypothalamus-pituitary-organ axes. These physical stressors modulate molecular pathways, including MAPK and NF-κB signaling, oxidative stress responses, and circadian rhythm regulation, thereby affecting reproductive and neuroendocrine functions. Their physiological and biochemical effects depend on exposure intensity, duration, and timing; these effects vary according to sex and species. Non-chemical or biochemical endocrine disruptors may exhibit adverse or beneficial effects, depending on exposure conditions, and can modulate major endocrine hormones and inflammatory mediators, supporting their therapeutic applications. Music therapy, cryotherapy, thermotherapy, phototherapy, and pulsed electromagnetic field therapies are used to reduce inflammation, enhance circulation, facilitate musculoskeletal recovery, treat neonatal jaundice, and manage diverse clinical conditions. This review aims to explain non-chemical environmental disruptors, their partially overlapping mechanisms that target the same hormonal, circadian, and cellular signaling pathways, and to discuss their potential therapeutic applications.

## 1 Introduction

EDCs are natural or synthetic compounds that mimic or interfere with hormonal signaling, affecting biochemical pathways, potentially causing adverse effects on development, reproduction, and metabolism. EDCs act as hormone agonists or antagonists, disturbing the hypothalamus-pituitary-adrenal (HPA) axis, hypothalamic-pituitary-gonadal (HPG) axis, and clarifying their molecular mechanisms is fundamental to assessing long-term health effects (1–3). EDCs are important risk factors for many diseases, including cancers (4–6). EDCs are accepted as a diverse group of substances and can be grouped as bisphenols (bisphenol A, bisphenol S, bisphenol F), phthalates [Di(2-ethylhexyl phthalates, dibutyl phthalate, di-isononyl phthalate], per- and polyfluoroalkyl substances (PFAS), polychlorinated biphenyls (PCBs), pesticides (DDT, atrazine), heavy metals (cadmium, lead, mercury), pharmaceuticals (ethinylestradiol, tamoxifen), and flame retardants (PBDEs) (7–15). There are also natural endocrine-disrupting substances (phytoestrogens, phytoandrogens, phytothyroids or phyto-goitrogen, mycotoxins, etc.) (16–22).

In addition to EDCs, some environmental factors can affect the endocrine system and trigger hormonal and metabolic changes that are considered non-chemical or environmental endocrine disruptors or physical stressors (23–25).

Environmental stressors can alter endocrine homeostasis, for instance, artificial light (23, 26–30), noise (31–38), temperature variations, global warming (39–43), and electromagnetic radiation (44–49). However, the potential health effects of electromagnetic fields (EMF) remain controversial, as epidemiological and experimental studies have produced inconsistent and often inconclusive results. Therefore, claims regarding EMF-related endocrine effects should be based on extensive and high-quality evidence (44, 46, 50). While some experimental animal studies have reported alterations in reproductive or stress hormones following EMF exposure, these studies are highly heterogeneous in terms of exposure conditions and endpoints, and their relevance to human health remains limited (44, 46, 51). The ICNIRP 2020 Guidelines systematic review concluded that only a small number of human studies have tested whether endocrine function is affected by RF-EMF exposure, and no consistent evidence of effects has been observed. Similarly, the SCHEER 2022 report highlights the need for further research, particularly in higher frequency bands of the RF spectrum, before firm conclusions can be drawn (52, 53).

Exposure to artificial light at night (ALAN) negatively affects circadian rhythm regulation, melatonin signaling, sleep regulation, reproduction, cell turnover, metabolic, and immune regulation (23–25). Hot and cold-water stress (a brief dip in an ice bath) affects the endocrine system (54) and produces health benefits (55). Classical endocrine disruptors mimic or interfere with the body’s hormones, disrupting the synthesis, transport, and metabolism of hormones that act through receptor binding or enzymatic interference (56). Physical non-chemical endocrine disruptors are thought to influence neuroendocrine signaling, neurotransmitters, the neuroendocrine-immune axis, oxidative stress, and enzymatic interferences, as well as the circadian clock, potentially affecting hormonal balance and metabolism. Preliminary methodological studies provide some indications, although limitations of the experimental protocols should be carefully considered (57).

This review aims to analyze current knowledge on non-chemical and biochemical environmental stressors and their molecular mechanisms underlying their endocrine effects. Unlike EDCs, which primarily target peripheral receptors and enzymes, non-chemical stressors initially affect central hypothalamic-pituitary regulation.

## 2 Adverse effects of environmental electromagnetic field exposure

A systematic review and meta-regression analysis provide evidence of a significant decline in sperm concentration between 1973 and 2011, primarily in North America, Europe, Australia, and New Zealand, while no significant decrease was observed in other regions (58). While multiple factors, such as endocrine-disrupting chemicals (EDCs), may contribute to this decline, the role of EMF exposure cannot be rejected but remains uncertain and requires further experimental studies to clarify its potential effects on fertility (59).

Animal studies suggest that EMF exposure may induce biological alterations depending on the duration and intensity of exposure. For example, exposure to 2.45 GHz Wi-Fi has been shown to induce oxidative stress in the testis after 4 hours, whereas longer exposures (8–24 hours) appear to activate cellular repair mechanisms. A review summarizing 18 studies reported that mobile phone RF-EMW exposure may reduce sperm motility, viability, and concentration; however, these findings were not statistically significant (60). Finally, the European Parliament’s 2021 report on the health impact of 5G concluded that information on potential risks of RF-EMF, including reproductive effects, remains limited (61).

Extremely low-frequency EMF (ELF-EMF) can affect brain function and induce cellular changes by activating the HPA axis, leading to the production of stress hormones, mainly cortisol (62, 63). Exposure to low-frequency EMF has been reported to influence brain function and activate the HPA axis, resulting in increased production of stress hormones such as cortisol and corticosterone (64). Long-term multifrequency EMF simulating the effects of 5G systems affected the functional activity of the HPA axis, and this exposure increased stress hormone secretion (65).

**Figure 1:**
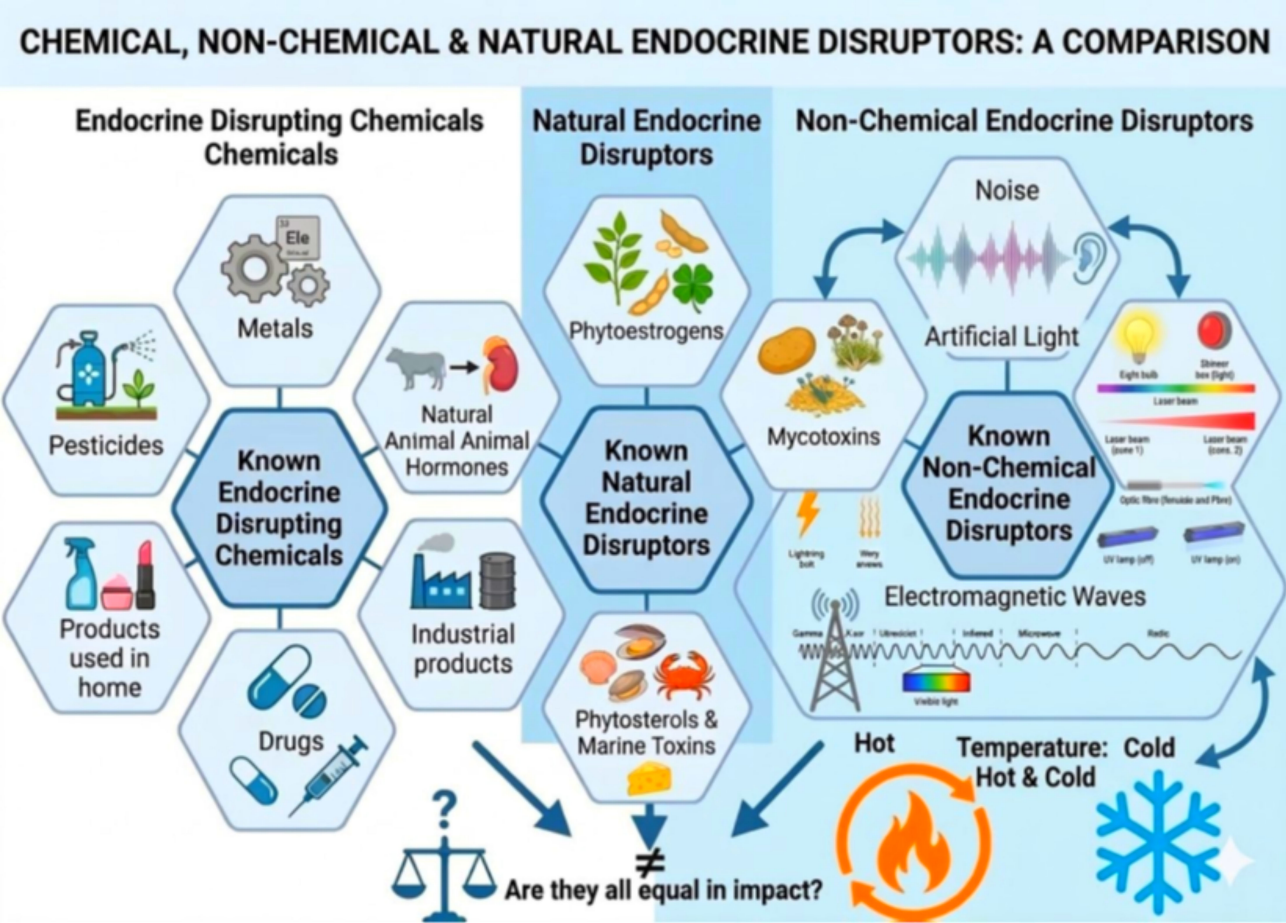
**(a)** Overview of non-chemical endocrine disruptors (ECDs), including noise, electromagnetic fields (EMF), artificial light at night (ALAN), and thermal stress (hot/cold). These physical stressors primarily affect the hypothalamus via auditory pathways, activating the HPA axis and disrupting endocrine function. Further research is needed to clarify EMF effects on humans and other organisms. **(b)** Classification of environmental stressors into non-chemical endocrine disruptors (noise, EMF, ALAN, thermal stress) and chemical endocrine disruptors (phthalates, BPA e.g., DEHP, parabens).

A comparative overview of EDCs and non-chemical or biochemical endocrine disruptors is presented in **Figure 1a**.

Long-term exposures of 1 hour/day for 52 days to 4 hours/day for 52 days were investigated. Extremely low-frequency electromagnetic field (ELF-EMF) at 50 Hz, 100 µT can decrease serum testosterone levels, spermatocyte counts, and motility, and induce changes in testicular tissue (66). EMF exposure may also influence long-term immune function (2–24 h/d up to 8 years). Exposure to ELF-EMF may affect the adaptive immune system and may increase innate immune responses (67).

Radiofrequency electromagnetic fields (RF EMF) range from 100 kHz to 300 GHz and may induce oxidative stress in both humans and animals. EMF exposure can induce oxidative stress in various cell types (68) and affect the metabolic and antioxidant enzyme activities (69, 70). EMF exposure has been suggested as one of the risk factors of Amyotrophic Lateral Sclerosis (ALS), which is the most common motor disease characterized by fatal prognosis (71, 72). Similarly, EDCs can also affect cognitive function, such as BPA, behavioral disturbances, and may cause ALS disease (73).

Overall, current evidence regarding the adverse endocrine and systemic effects of environmental EMF exposure is highly dependent on exposure parameters, such as frequency, intensity, duration, and organism.

### 2.1 Therapeutic applications of controlled EMF exposure

In contrast to environmental exposure, controlled pulsed electromagnetic field (PEMF) applications have been investigated, which may have beneficial effects on health. PEMF affects molecular signaling pathways, RANK, MAPK, NF-κB, adenosine receptors, calcium channels, bone morphogenetic protein-2 (BMP-2), and Wnt1 in osteoblasts and osteoclasts (74).

Low-frequency, low-energy pulsed electromagnetic fields (PEMFs) appear to modulate inflammatory pathways *in vitro* in joint and neurological disorders, although the exact neuroprotective mechanism remains unclear (75). PEMF therapy has anti-inflammatory effects, supports bone regeneration, and potentially benefits in fracture healing and wound repair in animal models (76, 77). Finally, complex magnetic fields offer potential in innovative therapeutic strategies (78) such as pain and injuries (76, 79).

PEMF treatment reduces inflammation, supporting bone tissue regeneration, *in vivo* and *in vitro* (80). It has been investigated that PEMF has potential therapeutic effects on bone fracture-related conditions and wounds in rats (81) and promotes early wound healing and myofibroblast proliferation in diabetic rats (82). PEMF therapy in human patients may have beneficial effects on mandibular fracture healing via increasing bone density and increasing new bone formation (83). PEMF treatment may reduce apoptosis and reactive oxygen species (ROS) levels, induce hypoxia/reoxygenation, and phosphorylate protein kinase B (Akt) and endothelial nitric oxide synthetase (eNOS), thereby protecting against ischemia/reperfusion-induced injury in rats (84) and promoting stem cell chondrogenesis (85). PEMF may accelerate wound healing in the skin of diabetic rats (86). Exposure to extremely low-frequency pulsed electromagnetic fields (ELF-PEMF) enhances the human dermal fibroblast proliferation, migration, and myofibroblast differentiation *in vitro*. These effects suggest that ELF-PEMF may involve dermal wound healing (87).

Chronic immobilization stress disrupts the HPA axis by elevating the corticosterone levels, while repeated electromagnetic stimulation and oxytocin help to restore HPA function (88).

Human exposure to environmental EMFs is typically chronic and difficult to control (89). On the other hand, therapeutic PEMF applications involve completely defined frequencies and duration (90). The seemingly conflicting adverse and beneficial findings may therefore reflect non-linear dose-response relationships and differences in experimental design rather than true contradictions (91).

All these observations highlight the complexity of EMF bioeffects and the importance of exposure conditions in determining outcomes.

## 3 Noise exposure as a non-chemical endocrine disruptor

Noise is recognized as a physical agent (92) and a major environmental pollutant that activates neuroendocrine stress pathways, leading to the elevation of stress hormones, including corticosterone, adrenaline, noradrenaline, and endothelin-1 (93). The primary target is the central nervous system, the hypothalamus, then hormone receptors or steroidogenic enzymes in the peripheral tissues, and then physiological and biochemical effects on the organism (94, 95), as shown in **Figure 1b**.

### 3.1 Effects of noise exposure on the HPA axis and HPG axes

Noise exposure rapidly activates subcortical auditory circuits, the HPA axis, and affects neuroendocrine and metabolic disturbances, causing the release of stress hormones such as corticotropin-releasing hormone (CRH) and adrenocorticotropic hormone (ACTH) (96), as well as catecholamines (95). Noise intensity effects of 95, 105, and 115 dB were tested on male rats, and the levels of ACTH, cortisol, and testosterone were investigated. Testosterone level decreased, but ACTH and cortisol levels increased to 115 dB (97). Aircraft noise can lead to the secretion of cortisol during sleep (98).

Marine noise pollution is accepted as strong noise that significantly interferes with the expression of HPA axis functional genes, including CRH, corticotropin-releasing hormone receptor 2 (CRHR2), and arginine vasotocin (AVT). Long-term stimulation of noise can induce brain, heart, and adrenal gland tissues’ metabolic disorders, which is accepted as posing a lethal factor (99). Chronic noise exposure dysregulates the neuroendocrine system, causing hyperactivity of the HPA axis, increases stress hormones, affects brain function, and may lead to Alzheimer’s disease-like neuropathological changes (100). Noise-related hearing loss induces the production of autoantibodies of Hsp70 and Hsp60, which are related to autoimmune disease and may cause non-Hodgkin lymphoma, and the HPA axis is involved in autoimmune diseases (36).

Noise stress may have negative effects on male sex hormones and reproductivity, but swimming modifies the effect of noise stress on the HPG axis in male rats (101). Noise decreased the 11-ketotestosterone level in males and increased it in females, inhibited gonadal maturation, and altered the expression of the key genes in the HPG axis in zebrafish. Therefore, noise may be considered a non-chemical endocrine disruptor in this model (102).

### 3.2 Noise-induced activation of stress, inflammatory, and metabolic pathways

Noise triggers also inflammatory pathways, first triggering the HPA axis, then affecting peripheral enzymes. For instance, it also activates NADPH oxidase, nitric oxide synthase, induces mitochondrial dysfunction, causes uncoupling leading to oxidative stress, and inflammatory responses (93, 103, 104). All these metabolic changes contribute to a range of disorders, such as vascular and cardiac damage, and noise-induced hearing loss (105, 106). Short-term aircraft noise activates the renin-angiotensin-aldosterone system, enhances glycolytic activity and glucose-6-phosphate, lactic acid, oxygen-independent ATP production, glycerol-3-phosphate, a metabolite linking glycolysis to lipogenesis, serotonin (5-HT), and significantly alters amino acid and lipid metabolism (107). Chronic exposure to 100 dB noise, 4–6 hours daily in Sprague-Dawley rats of both sexes, resulted in altered thyroid morphology, characterized by an increase in follicular diameter in both sexes, but TSH level is increased only in females (108). Wilson and Apawu demonstrated that noise has an impact on dopamine levels, and the deafening noise downregulates dopamine transmission (109).

### 3.3 Music exposure as a therapeutic neuroendocrine modulator

Most of the population accepts that music is a form of medicine, and music therapy can be used to enhance physical and mental health (110). The type of music affects the individual relaxation response: melody, rhythm, tonality (Mode/Tone), and frequency are important in relaxation. The researcher indicated that 528 Hz music can be listened to for relaxation purposes in humans. Music has different effects on human hormone secretion; for instance, high-frequency music plays a role in stress relief, while 528 Hz frequency music lowers cortisol levels and increases oxytocin levels (111). Music tuned to 528 Hz is widely recognized as a therapeutic frequency. Research evaluating its physiological effects through biomarkers in human saliva has demonstrated a significant reduction in cortisol and chromogranin A levels, alongside a notable increase in oxytocin. These findings suggest that 528 Hz frequency facilitates stress reduction and promotes emotional well-being (112). 432 Hz music is effective in decreasing salivary cortisol levels in patients undergoing tooth extraction (113). Music therapy is an alternative therapy method for attention-deficient hyperactivity disorder, which increases the serotonin level and decreases the cortisol level. This means that music improves stress-coping ability by changing stress hormone levels (114). Investigating the neuro-hormonal effects of 528 Hz sound waves reveals a measurable correlation between specific frequency exposure and increased testosterone production in rats. Specific frequency stimulation may modulate the hypothalamic-pituitary-gonadal axis, potentially leading to an upregulation of central testosterone levels and a reduction in anxiety-like behaviors in rats (115).

## 4 Light exposure as a non-molecular endocrine disruptor

### 4.1 Effects of artificial light at night and the HPA and HPG axes

Exposure to ALAN disrupts hypothalamic regulation of the endocrine system, especially affecting melatonin, corticosterone secretion via the HPA axis, and reproductive hormones via the HPG axis (28). The researchers exposed birds to constant light, then a normal light/dark cycle for 23 days, then waited for 12 days, and found that the HPA axis was not affected, and glucocorticoid receptor levels stayed the same (116). Exposure to ALAN disrupts melatonin secretion, leading to cortisol secretion during the night, disrupts adolescent circadian rhythm, which contributes to adverse behavior and cognitive outcomes, negatively affects moods, and increases stress and HPA axis activation (117). Monochromatic light affects the HPA axis by altering CRH-ACTH-corticosterone signaling, stress response, and metabolism. Green light increases muscle growth through GHRH-IGF-1, upregulation, blue light affects the thyroid axis (TRH-TSH-T4/T3), and affects muscle growth, reproductive function, and metabolic regulation (118).

ALAN affects the HPG axis and affects steroid synthesis, germ cell development, testis growth, and modulates hormonal regulation and reproductive physiology at low levels (119). Environmental light and chemical pollution can interfere with the photoneuroendocrine system and alter reproductive rhythms in humans and wildlife (120). ALAN increases the activation of the reproductive endocrine process via the HPG axis in tree sparrows (121). Small changes in the nocturnal light intensity affect the reproductive physiology of wild organisms (119).

### 4.2 Artificial light-mediated modulation of hormonal homeostasis

Light is essential for most types of living organisms to regulate biological responses, gene expression, circadian rhythms, sleep-wake cycles, and metabolic activity. Researchers examined these biochemical and physiological effects under varying light intensities, durations, exposure timings, and wavelengths, as well as hormonal homeostasis (122, 123). Light affects nocturnal and diurnal animals differently (124).

ALAN can directly affect endocrine signaling and disrupt circadian regulation, decrease melatonin production and increase inflammatory responses, elevate circulating stress hormones, and act as an environmental disruptor (23). ALAN has been linked to adverse metabolic effects by affecting the HPA axis (120, 121), and the HPG axis, resulting in body lipid accumulation and increased levels of reproductive hormones, gonadotropin-releasing hormone 1, luteinizing hormone, follicle-stimulating hormone, and 17-beta-estradiol in humans (125), ALAN can cause sleep disturbances and increase the risk of cancer, including breast cancer (126, 127), adversely affect mental health, and elevate the risk of hypertension, diabetes, obesity, cardiovascular disease, and psychiatric disorders, including anxiety and depression in students (128). Exposures to ALAN from smartphone screens are blue-enriched light, which disrupts circadian endocrine regulation and suppresses melatonin secretion (129). Wearable sensing technologies and smartphones allow continuous monitoring of physiological and behavioral markers affecting circadian rhythm, hormonal fluctuations, metabolic regulation, and cause endocrine disruptions (130). In a very recent study, it was suggested that smartphone-based interventions can support return to work by monitoring stress, sleep, and behavior, which influence HPA and HPG axes activity and contribute to endocrine regulation recovery (131).

Dim light at night (dLAN), a low-intensity form of ALAN, has been shown to decrease locomotor activity in African pygmy mice and disrupt glucose and fat metabolism (132), circadian rhythm, sleep, and cause fattening in zebra finches in a sex-dependent response (116). Daytime exposure to monochromatic blue light modulates effects on the endocrine rhythm, and the excretion of electrolytes increases (133). Continuous light and dark exposure affect mitochondrial complexes, membrane-bound transporters, inflammation, autophagy, and neurodegeneration in rats (134).

### 4.3 Effects of light intensity on metabolism

High intensity light (HI) combined with circadian disruption HI-CD increases serum glucose levels and hepatic triglyceride levels, body weight, adipocyte size, β-cell mass, and insulin resistance, which causes obesity in both the low-fat diet and the high-fat diet (135) while strongly decreasing melatonin levels in Arctic charr (136). Morning exposure to HI decreases cortisol levels and decreases body temperature (137). Dual light intensity reduces plasma corticosterone levels and affects tryptophan hydroxylase, the rate-limiting enzyme of serotonin, and tyrosine hydroxylase, the rate-limiting enzyme of dopamine synthesis in humans (138).

### 4.4 Light wavelengths and effects on hormones and signaling pathways

Short-wavelength, low-intensity blue light has negative effects on children, affecting the circadian system and disrupting melatonin secretion, leading to circadian misalignment and impaired sleep quality (139). In contrast, longer wavelengths and red light have a positive effect on the gonadal development of the Japanese quails (140). In humans, UV-B exposure triggers the conversion of 7-dehydrocholesterol to vitamin D3, which is hydroxylated in the liver and kidney to form 1, 25-dihydroxyvitamin D (calcitriol) (141). High light intensity upregulated MAPK-dependent melanin synthesis by activating the tyrosine metabolic pathway in an edible medicinal mushroom (142). Light intensity induces oxidative stress and inflammation, causing cell death and retinal neurodegeneration in mice (143).

Circadian rhythm proteins and hormones have a direct effect on inflammatory responses, pathophysiological pathways, and immune modulatory effects (144).

Calcitriol regulates calcium phosphate homeostasis, immune function, ovarian steroidogenesis, and androgen production (145). UV-B exposure has been shown to elevate circulating sex steroid levels both in mice and humans, indicating that UV-B exposure induces the skin-brain-gonad axis and has photobiological effects on sexual behavior and reproductive endocrine function (146). Morning exposure to short-wavelength light, approximately 470 nm, enhances the cortisol awakening response (CAR) (147). The circadian rhythm of cortisol is essential for metabolic balance and stress homeostasis, which is disrupted by night-shift work (148). Wavelength-dependent effects vary between humans and animals owing to distinct evolutionary adaptations in their photobiological signaling mechanisms, molecular pathways, and endocrine systems (149).

### 4.5 Melatonin as a key modulator of light-induced endocrine regulator

Although melatonin suppression is mentioned in earlier sections, this section focuses on molecular mechanisms and systemic endocrine roles.

Melatonin is the main hormone that induces sleep and has circadian effects on metabolism (150), and exposure to bright light, approximately 424, 460, and 480 nm, and ALAN is associated with melatonin suppression (151). Thus, melatonin deficiency is known as darkness deficiency (152). The pineal melatonin interacts with the clock genes BMAL1/BMAL2, CLOCK, CRY1/CRY2, and PER1/PER2/PER3, which are related to circadian rhythm, can control cellular biology, biochemical pathways responding to light changes in our environment, and are also molecular targets for chemotherapy and immunotherapy (153–155). Melatonin regulates circadian rhythm, acts as an antioxidant and free radical scavenger, protects cells from oxidative stress, prevents atherosclerosis, and treats cardiovascular diseases (156), body temperature, reproductive system, endocrine system (157), physiological system, hormone secretion, and metabolic homeostatic control. Melatonin mitigates cadmium-induced ferroptosis in spermatogonia by modulating ferritinophagy and regulating iron homeostasis (158). On the other hand, glioblastoma is the most common malignant primary brain tumor, which is linked to circadian clock dysregulation. The core clock regulators BMAL1 and CLOCK promote stem-cell maintenance and pro-tumorigenic microenvironment (159).

Melatonin is a multifunctional master regulatory molecule in poultry that affects reproductive and productive performance, muscle growth, milk yield, and the metabolic and endocrine systems via the pineal-melatonin axis. Farmers use melatonin implants for sheep (160) for reproduction and sexual behaviors in goats (161)and rabbits (160, 162).

### 4.6 Therapeutic applications of light exposure

Light, especially ALAN, has endocrine-disrupting effects (28), but wavelength-specific light exposures can exert therapeutic benefits on humans (163). Phototherapy may have beneficial effects on depressive symptoms and neurodegenerative processes and can be used in migraine and fibromyalgia (164). Psoriasis is a chronic autoimmune disease, and phototherapy is widely used with UVB, psoralen UVA (PUVA), pulsed dye laser (PDL), photodynamic therapy (PDT), intense pulsed light (IPL), and light-emitting diodes (LED) (165). Blue light has two major effects on human health. The first one is beneficial and can be used for the therapy of acne vulgaris, psoriasis, and atopic dermatitis. The second one is detrimental, which can increase radical production and induce skin hyperpigmentation, accelerate skin aging, cause DNA damage, and activate apoptotic pathways (166). In the therapy of neonatal jaundice, blue light has clinical benefits (167). Green light has health benefits, including improved sleep quality, decreased anxiety, and chronic migraine (168). Green light increases glutamatergic neurons; red light increases GABAergic neurons. Greenlight increases glutamatergic sensitivity to noxious stimuli in the vLGN red light enhances nociception via GABAergic activation. Light color differentially modulates pain by glutamatergic and GABAergic subpopulations in the vLGN, offering potential targets for precise neuropathic pain therapy (164, 169). Green light significantly extended the lifespan of Drosophila melanogaster, whereas blue light reduced it (169). Green light therapy reduces knee joint pain and induces lipidomic alterations in osteoarthritic rats (170). The application of red light 660 nm has been shown to have several health benefits, such as reducing pain in neuropathies; on the other hand, visual application exacerbates migraine headaches (171). 660nm red light has beneficial effects on allergic rhinitis and can be used in rhinophototherapy (172). Twice-daily exposure of the retina to repeated low-level red light (RLRL) is a non-pharmaceutical method used to slow or prevent myopia (173).

Circadian rhythms are essential for health, but modern lifestyle factors, such as using artificial light, solar variations, and EMFs, can disrupt and alter melatonin and cortisol (174).

## 5 Heat and cold exposures as an endocrine disruptor

### 5.1 Effect of heat exposures on the HPA and HPT axes

Heat exposure and ozone were applied to male mice to investigate the heat effect on the HPA axis and sympathetic-adrenal-medullary (SAM) axis. This exposure was found to mediate the release of ACTH, epinephrine, CORT, and Hsp70 in C57BL/6J male mice (175). Heat stress activates the HPA axis and induces depressive disorders, overexpression of heat shock proteins and glucocorticoid receptors, and suppresses brain-derived neurotrophic factors (BDNF) on humans (176). Heat stress, 40 ˚C environment, activates the HPA axis, hypothalamic, pituitary, testis (HPT) axis, expressing energy metabolites, amino acid neurotransmitters, and monoamine neurotransmitter pathways in male Sprague-Dawley rats (177). Heat stress induces oxidative stress, decreases the synthesis of arginine and glutamate, and downregulates GABA and BDNF, which triggers the HPA axis. Inflammatory responses, oxidative stress, and the activation of the HPA axis cause anxiety in 20 college students (178). Poikilotherms and homeotherms have different, well-defined metabolic responses to temperature differences, and they need to be discussed, but there are potential limitations associated with extrapolating results from poikilothermic species (179).

Heat stress affects energy metabolites and metabolic hormones, causes nutritional and physiological stress, and activates the HPG or HPA axis, resulting in decreased pregnancy rates in cows (180). Heat stress is accepted as a significant problem in the milk industry. Heat stress can affect physiological and biochemical parameters, fat production, milk yield, and composition, milk and blood metabolites in different breeds such as Brahman, Angus cattle (181). Lim et al. explored non-invasive physiological responses to heat stress on milk production and milk protein content in Holstein and Jersey cows in Korea. They found that milk production is not changed, Holstein cows have less tolerance to heat, and the respiratory rate is increased and higher body surface temperatures of rumen (RST), rectal temperature (RT), and udder surface temperature (UST) than Jersey cows (182). Heat stress during summer reduces kisspeptin expression and, under these conditions, downregulates the HPG axis, leading to summer infertility in domestic sows (183). Heat stress for 90 days increases stress hormones Hsp70, CORT, estradiol, luteinizing, and insulin hormone; insulin hormone, FSH, and prolactin increase; GnRH and T4 showed no difference in female rats (184).

### 5.2 Effects of cold exposure and HPT and HPG axes

Thyroid hormones play a key role in regulating body temperature, and in cold exposure, the hypothalamus-pituitary-thyroid (HPT) axis is activated in the cold, activating TRH-producing neurons, which increases TH in humans (185). Cold exposure activates the HPT axis and decreases T3, TRH, acute stressors, and corticosterone in male rats (186).

During the hibernation in cold, GnRH, LH, FSH, and progesterone significantly decreased, but testosterone level increased after a 135-day experiment in female lizards (187). In cold temperature zones, seasonal reproduction is regulated by the mediobasal hypothalamus to the HPG axis, but the regulatory elements are not well understood in male quails (188).

### 5.3 Temperature variations and global warming

From prokaryotes to eukaryotes and from invertebrates to vertebrates, organisms have developed a variety of adaptive mechanisms to survive across a wide range of temperatures (189). Temperature variations have important effects on the endocrine system, hormone secretion, receptor sensitivity, and physiological response (190). Temperature stress, heat, or cold activates the HPA axis (178, 191). Heat stress alters physiological and biochemical parameters by dysregulating the HPA axis, characterized by increased levels of cortisol, catecholamines, and Hsp70. And decreases body weight (192). In hot temperatures, corticotropin-releasing hormone (CRH) is secreted from the hypothalamus. The CRH regulates cortisol secretion in medaka (193). Then, cortisol influences metabolism, immune response, and neurobiology; it is an important factor in adaptation to acute stress (194). Climate can affect the levels of prolactin, progesterone, cortisol, estradiol, follicle-stimulating hormone, and luteinizing hormone (39). As the climate changes, with both extreme heat and sudden cold, the key point is the ability to survive, reproduce, and maintain a stable population over time (195). Heat stress due to climate warming decreases luteinizing hormone and follicle-stimulating hormone, affecting spermatogenesis and testosterone levels in male adolescents (196), whereas no correlation was found between ACTH and cortisol levels in human hypothermia cases (197).

### 5.4 Hormonal, oxidative, and reproductive impacts of heat stress

Heat stress activates the HPA axis, increases the Heat Shock protein 70 (198), increases the production of plasma triiodothyronine, but decreases thyroid-stimulating hormone (199), and increases the cortisol and aldosterone levels (199, 200). Heat stress has significant effects on the neuroendocrine system and may lead to organ damage (201). Heat stress causes irregular menstrual cycles, hormonal imbalances, reduced oocyte quality, and decreased ovarian function in maladapted women (202). Exposure to heat causes a rise in testosterone in maladapted miniature pigs (203). Prolonged exposure to high temperature resulted in a decrease in the activities of superoxide dismutase, catalase, 17β-hydroxysteroid dehydrogenase-3, and glutathione levels, but an increase in lipid peroxidase, caspase-3, testicular heat shock protein 72, heat shock protein-1, and corticosteroid concentration in the maladapted male rats (204). Heat stress inhibits the secretion of luteinizing hormone and gonadotropin in dairy cows (205) and estrogen levels in domestic sows (183). Heat exposure caused oxidative stress and increased levels of inflammation, plasma cortisol, C-reactive protein, NF-κB, malondialdehyde, nitric oxide, but decreased the alkaline phosphatase levels relative to the control in rats (206). Acute heat exposure increases the ACTH, cortisol, IL-2, and IL-12 in rat serum (207). Hot thermal stress and cold water affect cortisone and testosterone levels in young men (54).

### 5.5 Neuroendocrine and metabolic mechanisms triggered by cold exposure

Cold exposure may result in increased free fatty acid (FFA) utilization compared to production, leading to decreased FFA levels and enhanced insulin sensitivity in obese rats (208). Cold exposure decreased the anorexigenic brain-derived neurotrophic factor (BDNF) messenger RNA levels and increased the growth hormone-releasing hormone (GHRH) levels, contributing to the development of obesity and impairment of glucose homeostasis in C57BL/6 mice (209). Upon cold exposure, noradrenalin and plasma triglyceride levels increased, and activated brown adipose tissue, potentially mediating obesity in young men (210). Cold-induced energy metabolism in humans (211). At lower temperatures, the expression of the hormone gene gh1 and its related receptors, GHRα, GHRβ, IGF1Rα, and IGF1Rβ, is reduced in various organs of fish, such as skin, brain, and eye (212).

Cold stimulates the hypothalamo-pituitary-thyroid axis and the release of thyrotropin-releasing hormone (TRH) and catecholamines (213). Kovaničová et al. reported that ice water swimming increased parathyroid hormone and TSH levels and decreased T3 and T4 levels following a 15-minute winter swim. After cold exposure, T4 and T3 decreased in healthy factory workers (211). Cold exposure exerts neuroprotective effects, increases neurotrophic factors and mitochondrial markers, such as cytochrome c oxidase subunit I (COXI), A kinase anchor protein 1 (AKAP1), RNA-binding motif protein 3 (RBM3), and these signaling mechanisms depend on the duration and intensity of cold exposure (213). Seasonal temperature fluctuations significantly affect thyroid hormone levels, lower in summer but higher in winter, in elderly women (214).

Cold exposure may trigger inflammatory responses in the reproductive system and lead to disruptions in sex hormone levels in female mice (215). Xu et al. reported changes in uterus morphology and an increase in progesterone level, and a decrease in the ER level in female rats due to cold exposure (216).

### 5.6 Thermal hydrotherapy and cryotherapy: psychological effects and therapeutic applications

Heat, 36 °C, and cold, 20 °C, can be used as hydrotherapy to minimize fatigue or for post-exercise recovery in athletes (217). There are various types of hydrotherapy, including thermal care, balneotherapy, and spa treatments. It is found that warm water has positive effects on blood circulation and blood pressure, as well as post-surgical physical activity (218, 219). Thermal rehabilitation, hydro-kinesitherapy, is found to be beneficial for disabilities (220), spa therapy for rheumatology (221), and knee osteoarthritis (222).

Cryotherapy is commonly used for the treatment of musculoskeletal injuries, and novel applications involving exposure to 15 °C for 3–6 hours (223). The role of cryotherapy in human muscle regeneration following injury remains unclear. On the other hand, hot water therapy has been shown to upregulate interleukin-10 and heat shock proteins (60 min 42 ˚C), thereby enhancing muscle regeneration. In contrast, cold water therapy at 15 °C for 12 min does not show similar results (223).

## 6 EDCs induce specific metabolic pathways or molecules, like non-molecular endocrine disruptors

**Figure 2:**
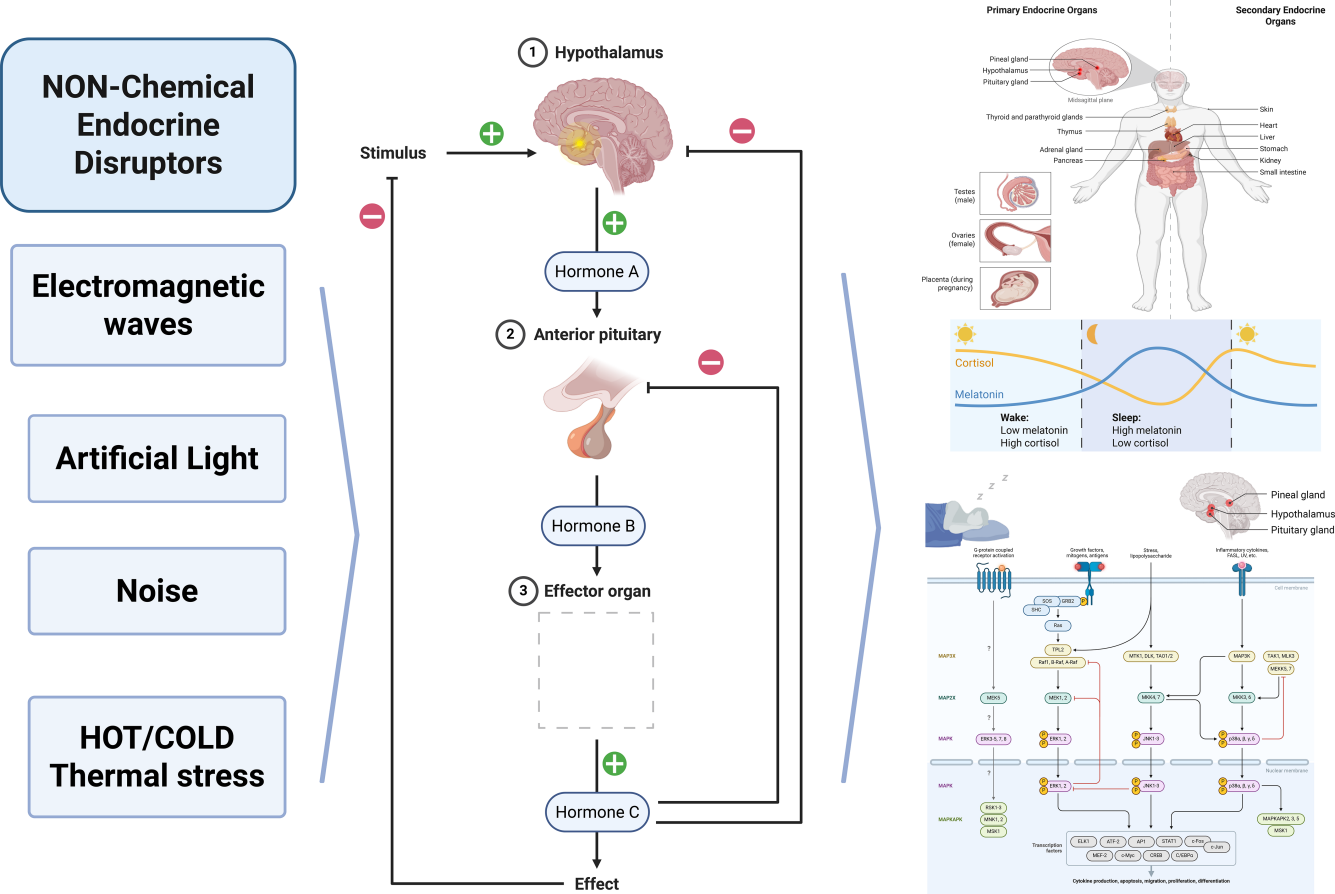
**(a)** EDCs bind enzymes in peripheral tissues, interact with nuclear receptors and steroidogenic or metabolic enzymes, and are metabolized into secondary metabolites. Their tertiary effect involves disruption of hypothalamus-pituitary-organ axes (e.g., HPA, HPT, HPG), subsequently affecting molecular and cellular endocrine targets in peripheral organs. **(b)** The figure shows DEHP and its active metabolite MEHP. DEHP inhibits beta-hydroxy steroid dehydrogenase, disrupting the HPA axis and increasing stress hormones like ACTH and cortisol. This imbalance induces oxidative stress and activates the MAPK pathway, leading to anxiety, reduced testosterone synthesis, and decreased sperm count and motility.

EDCs are a group of chemical and biochemical substances with different structures and characterized by hormone-like effects (4, 224, 225). The study highlights that prenatal exposure to specific EDCs significantly modulates the cord blood transcriptome, specifically affecting 39 metabolically relevant transcription factors and key signaling pathways such as insulin and IL-6. These findings provide a critical biological link, suggesting that transcriptional alterations at birth may predispose individuals to metabolic disorders later in life (226). Research indicates that low-dose BPA exposure triggers adrenal steroidogenesis by activating the JNK/c-Jun signaling pathway and upregulating Cyp11a1 expression, potentially linking this endocrine disruption to metabolic and neuropsychiatric disorders (227). Exposure to these substances disrupts the endocrine system, affects the immune system, gene regulation, induces epigenetic modifications, and affects related metabolic and neuronal pathways in the exposed organism, leading to various reproductive and developmental disorders and health problems (228–230). Shown in **Figure 2a**.

The best-known effects of EDCs are on the endocrine system, especially on the reproductive system (228), such as for BPA, which has estrogenic effects, affects testosterone production (231), circadian rhythm, such as the peripheral clock system (232), and circadian clock genes Bmal1, Per2, Rev-Erb alpha (233). Up to now, it has been investigated that 40 environmental chemicals dysregulate circadian rhythm, and these chemicals are classified under 6 groups, such as steroid hormones, metals, pesticides, biocides, polychlorinated biphenyls, neuroactive drugs, cyanobacterial toxins, and BPA (234). Phthalates such as DEHP are found in plastic toys, medical tubing, consumer products, medical devices, and plastic bottles, modulating the hormones associated with sleep and depression (232). Shown in **Figure 2b**.

BPA exposure would decrease melatonin and the melatonin receptor levels (235). BPA exposure is linked to reduced vitamin D levels, which are associated with increased cardiovascular risk in the elderly population (236). Bisphenol S is associated with the progression of glioblastoma (237). Low-dose BPA activates Cyp11a1 gene expression and stimulates corticosterone secretion in the adrenal through the JNK signaling pathway (227). Kaimal et al. examined the sex and dose-dependent effects of BPA, DEHP, and the combination of these EDCs on corticosterone levels. In this research, it has been investigated that male and female offspring prenatally exposed to these EDCs affected the HPA axis and increased the corticosterone levels (238). Similarly, the neuroendocrine disruptor dexamethasone affects thyroxine (T4), triiodothyronine (T3), and increases thyrotropin (TSH) levels but decreases epinephrine and norepinephrine and elevates dopamine levels (239). The fungicide methyl thiophanate affects adrenal pituitary hormone levels, and the thyroid gland increases adrenaline-producing cells, and affects adrenaline and noradrenalin levels (240). Exposure to low-dose BPA induces oxidative stress, upregulates the renin-angiotensin system, and aggravates hypertension, affects antioxidants, lipid abnormalities, and TCA cycle enzymes (241). Phthalates and bisphenols affect the phosphoglucose isomerase, phosphofructokinase, and other glycolytic enzymes, such as hexokinase and pyruvate kinase, in Spodoptera littoralis (242). Exposure to phthalates affected the HPA axis in female rodents (243). DEHP also affects the circadian rhythm and cytochrome p450 gene expression (244). EDCs can affect circadian, reproductive, behavioral, and metabolic rhythms (155). Bisphenols, parabens, and triclosan can affect the female reproductive system and cause polycystic ovary syndrome and chronic inflammation (245). EDCs inhibit antioxidant enzyme activities (246, 247). Flutamide is an EDC that affects the MAPK pathway and mitigates testicular damage in Sprague-Dawley rats (248). BPA effects NF-κB pathway and adenosine receptors (249), also alters molecular G protein-coupled pathways, estrogen-related receptor gamma pathway, homeobox-containing gene pathway, and bone morphogenic protein 2 (BMP2), (BMP4) (250). Exposure to this BPA is also a risk factor in the development of neurodegenerative diseases such as Parkinson’s and ALS (73). BPA, DEHP, diethylstilbestrol, cadmium, and 2, 3, 7, 8-tetrachlorodibenzo-p-dioxin can alter the evolutionarily conserved Wnt signaling pathway, which affects cell migration, polarity, neural formation, organogenesis, and embryonic development (251, 252). Sasaya et al. investigated BPA, p-nonylphenol, and tributyltin chloride endoplasmic reticulum stress-associated apoptosis in PC12 cells (252). BPA upregulates heat shock protein 70 (253). Vinclozolin is a dicarboximide fungicide and EDC that causes endothelial injury via eNOS/Nox4/IRE1α signaling in prolonged exposures (254). Butyl benzyl phthalates affect lipid metabolism and may cause non-alcoholic fatty liver disease (255).

### 6.1 Mechanisms by which EDCs exert their effects

The EDCs, firstly, exert their effects by directly binding to the nuclear receptor or enzyme, acting like a substrate of the enzyme or transcriptional activators or co-activators of receptor hormones (256, 257). Secondly, the EDC metabolite may be more active than the EDC themselves, as is the case with the DEHP molecule; MEHP is a more active endocrine disruptor (258). As shown in **Figure 2a**, DEHP and its active metabolite MEHP inhibit enzymes and are involved in the HPA axis, resulting in various metabolic changes. Thirdly, they exert their effects by acting via the hypothalamus-pituitary-organ axes as (1, 259, 260) as seen in **Figure 2b**. The direct comparison of EDCs and non-chemical endocrine disruptors’ characteristic properties is given in **Table 1**.

**Table 1:** Comparison of chemical, biochemical, and physical endocrine disruptors.

Disruptor type	Primary endocrine molecular targets	Key hormonal effect
Non-chemical		
Noise	HPA axis, catecholaminergic system Oxidative stress, NADPH oxidase, NOS	Cortisol increase, ACTH, adrenaline, Decreased testosterone, Oxidative stress, altered glucose and lipid metabolism
Music Therapeutic	HPA axis, Serotoninergic system	Cortisol decreased, oxytocin and serotonin increased
Electromagnetic fields	HPG axis, Circadian rhythm, oxidative stress pathways	Oxidative stress and the immune system are altered
Pulsed electromagnetic fields Therapeutic	MAPK, NF-κB BMP-2, Wnt signaling	Reduced inflammation, increased tissue regeneration, and wound healing
Artificial light	Pineal gland, circadian clock, genes (BMAL1, PER, CRY), HPA, HPG axes	Decrease in melatonin, increase in cortisol, and circadian misalignment
Light therapy Therapeutic	Circadian rhythm, neurotransmitter balance	Hormonal regulation, analgesic effects
Thermal stress Hot	HPA axis, thyroid axis	Increase cortisol, aldosterone, decrease LH, FSH
Thermal stress Cold	HPT axis, brown adipose tissue	Increase energy expenditure, altered thyroid hormones
Thermal therapies Hydrotherapy, cryotherapy	Neuroendocrine-immune interaction Inflammatory pathways	Anti-inflammatory, tissue recovery, musculoskeletal rehabilitation
Chemical		
Endocrine-disrupting chemicals: e.g., BPA, DEHP, parabens, phthalates	HPA, HPG, HPT axes, circadian genes, MAPK, NF-κB, Wnt	Hormonal imbalance, circadian disruption, oxidative stress, metabolic disruption

The situation is slightly different with non-chemical endocrine disruptors. Although they exert similar effects to endocrine disruptors and affect the same metabolic and signaling pathways, non-chemical endocrine disruptors exert their effects by acting on the HPA and HPG or HPT axis, as mentioned in the previous sections.

## 7 Methodology

This study was designed as a narrative review with a structured literature selection approach to synthesize current evidence on chemical or biochemical and physical environmental endocrine disruptors, including electromagnetic fields (EMFs), and their DNA, endocrine, biochemical, and physiological effects. A comprehensive literature search was conducted in PubMed, Scopus, and Web of Science using combinations of keywords with Boolean operators (AND, OR) to optimize the retrieval of relevant studies (261). Approximately 480 articles and reviews were initially screened, of which 267 were included in the narrative synthesis. Weight was based on studies published in the last five years to ensure that the review reflects the most recent evidence.

The screening and selection process was conducted in a structured and transparent manner. Duplicate records were removed, titles and abstracts were screened for relevance, and full-text articles were assessed for eligibility based on predefined inclusion and exclusion criteria. Studies were eligible for inclusion if they were peer-reviewed, involved human, animal, or *in vitro* designs, and investigated chemical, biochemical, physical, or non-chemical environmental factors affecting endocrine, molecular, biochemical, or physiological systems. Exposure parameters, including frequency, field strength, and duration, for EMF studies, were required to be clearly defined. Mechanistic, experimental, observational, and interventional studies were all considered, and single experimental reports were included when they provided biologically plausible mechanistic insights or highlighted heterogeneity. For animal studies, experiments reporting statistically significant outcomes were prioritized to ensure biological relevance.

The methodological quality and potential risk of bias of the included studies were assessed using standardized criteria appropriate for the study design, including sample size, exposure characterization, and outcome measurement reliability. Biological responses to environmental exposures were interpreted in the context of interindividual variability, including genetic, epigenetic, sex-, age-, and anthropometric-related factors, and overgeneralization was avoided. Priority was given to the inclusion of the most recent review articles and original investigations to provide a current and comprehensive synthesis of evidence.

Exclusion criteria included non-peer-reviewed articles, conference abstracts without full data, studies lacking adequate exposure characterization, and opinion-only publications without primary or synthesized evidence. Both beneficial and adverse effects were considered to ensure balanced and transparent evidence integration across human, animal, and *in vitro* studies.

## 8 Future directions in research on EDCs and non-chemical environmental stressors

Research on EDCs and non-chemical environmental stressors is likely to grow significantly in the coming years. New technologies such as sensors, automated monitoring systems, and artificial intelligence are making it possible to detect and analyze both chemical or biochemical and physical endocrine disruptors in different environments. Combining environmental, physiological, and molecular data may help improve predictive models, identify cumulative and combined effects, and support more personalized risk assessment approaches.

New tools such as wearable nanosensors, smart textiles, and smart eyewear that can detect ambient EDC levels may also strengthen preventive healthcare. In different ecosystems, monitoring microplastic-associated EDCs together with environmental factors such as temperature and salinity can improve our understanding of the link between climate change and pollution.

Exposure patterns are becoming more complex and are likely to become a major focus of research in the very near future, particularly regarding chemical and biochemical mixtures, microplastics, light and noise pollution, and electromagnetic fields. Future regulations should therefore focus on cumulative stress rather than single substances.

## 9 Conclusion

Environmental non-chemical disruptors, noise, EMF, ALAN, and extreme temperatures, affect the endocrine system through the HPA, HPG, and HPT axes, depending on type, duration, and timing of exposure. Noise and EMF disrupt stress hormone secretion, neurotransmitter pathways, and metabolic homeostasis, whereas music and controlled PEMF may induce short-term physiological responses under controlled conditions, reduce inflammation, and support tissue regeneration. The effects of light depend on wavelength, duration, and timing of exposure. ALAN and blue light have adverse effects, but red light can be beneficial. Extreme thermal exposures elevate stress hormones, such as cortisol and catecholamines, while controlled cold or heat therapies can promote adaptive and therapeutic effects.

EDCs or their active metabolites exert negative effects by binding hormone receptors or enzymes, altering HPA, HPG, and HPT signaling, transcription, metabolism, and reproductive pathways, thereby negatively affecting health. Both chemical and non-chemical endocrine disruptors thus act in an axis-specific mechanism, modulating circadian and signaling pathways, and thereby significantly influencing endocrine and metabolic health.

Importantly, the integrative approach of this review considers multiple environmental exposures that often co-occur in real life and provides a broader perspective on endocrine disruption than studies focusing on single exposures. The observed uncertainty does not reflect a lack of rigor but rather the inherent heterogeneity of the evidence. Future research should focus on standardized exposure characterization, combined exposure scenarios, and long-term outcomes to support evidence-based public health guidance.
