# Supplementary figures and images for: Non-chemical, chemical, and biochemical, endocrine disruptors: biphasic health effects and pathophysiological insights
Source: Front Endocrinol (Lausanne). 2026 May 8;17:1813234. doi: 10.3389/fendo.2026.1813234 (PMC13193988; doi:10.3389/fendo.2026.1813234)

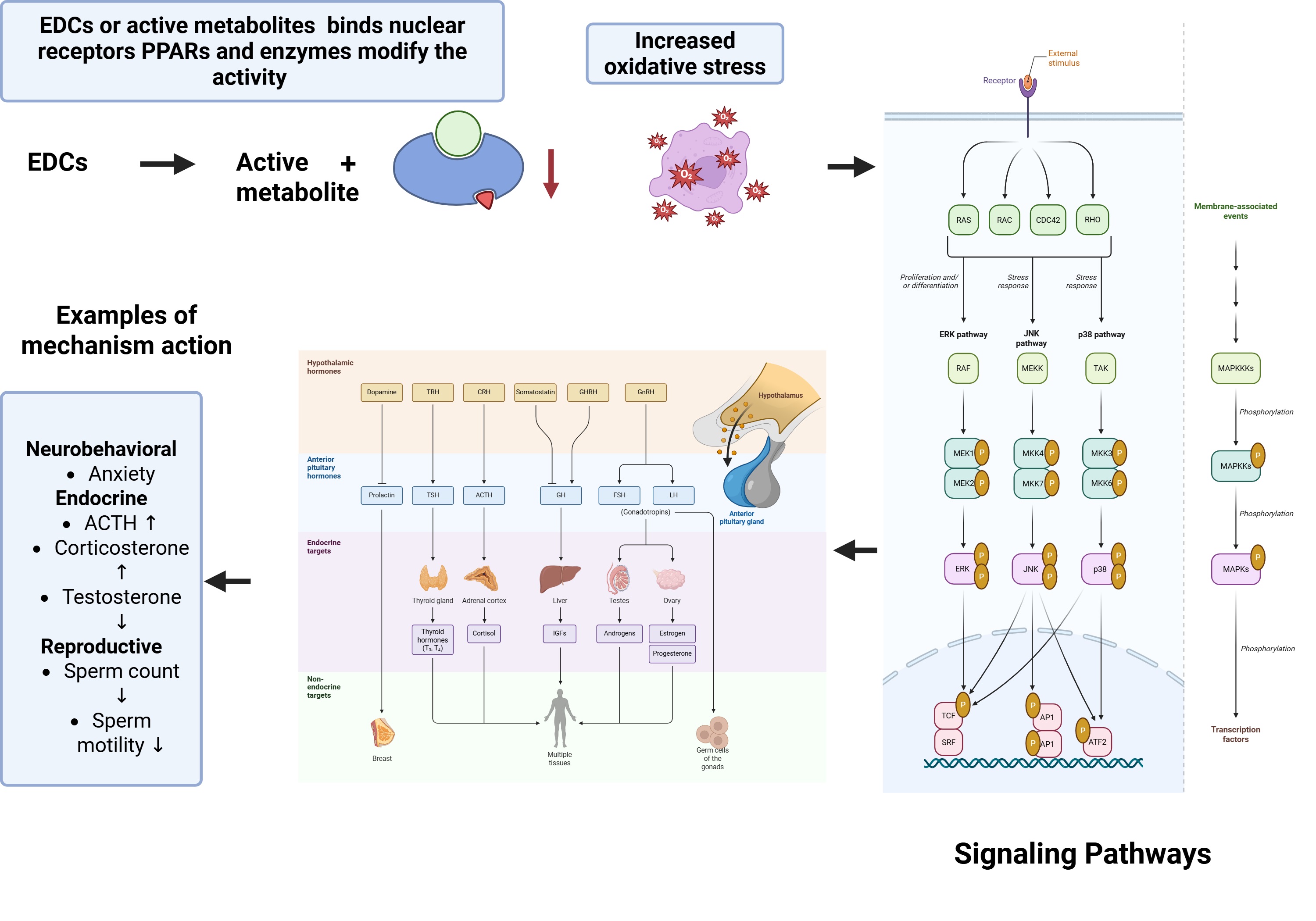

Supplement: Supplementary file 1 [file Image1.jpeg]

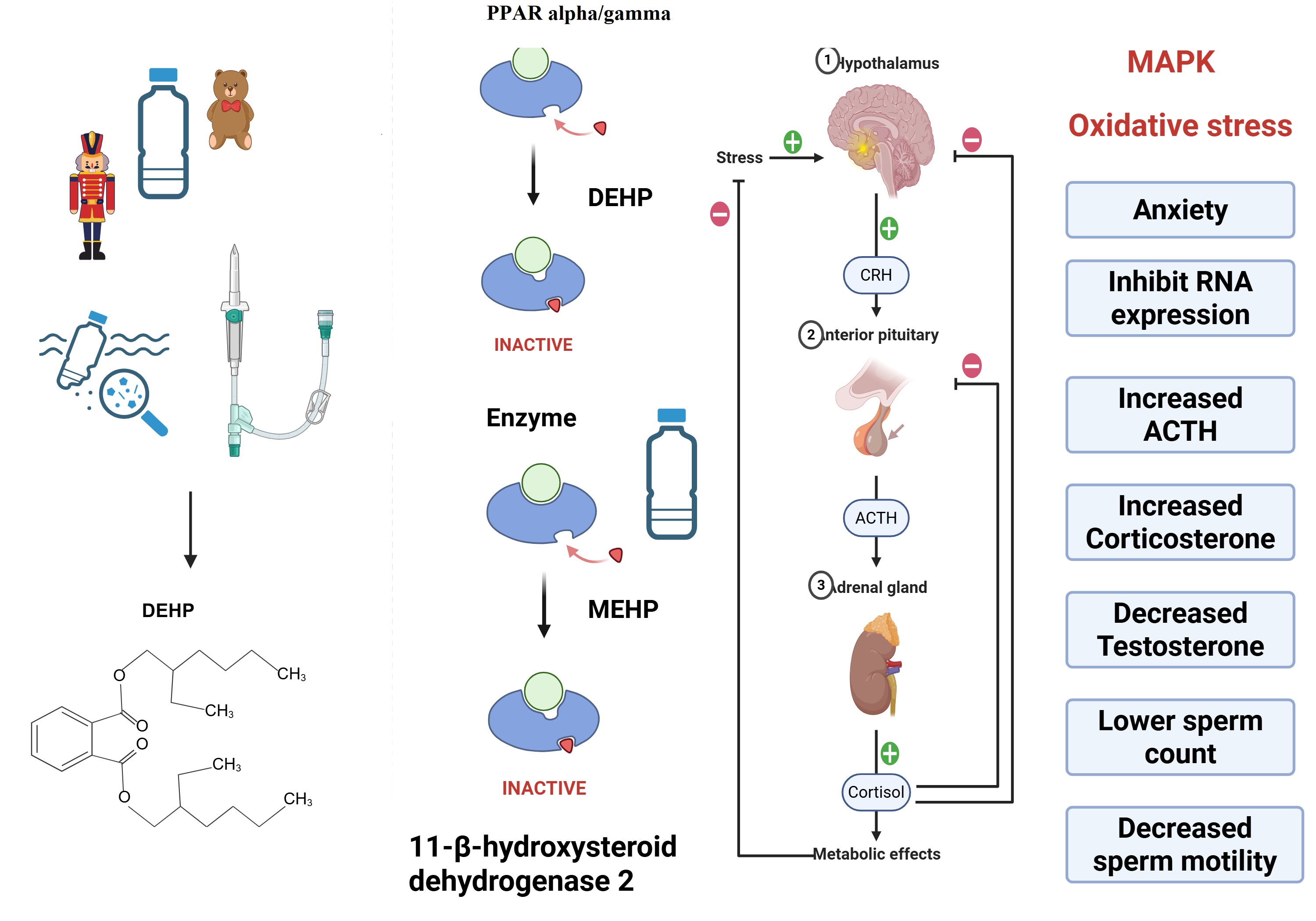

Supplement: Supplementary file 2 [file Image2.jpeg]
